# The Association between Allergic Rhinitis and COVID-19: A Systematic Review and Meta-Analysis

**DOI:** 10.1155/2022/6510332

**Published:** 2022-09-28

**Authors:** Cong Xu, He Zhao, Yuwan Song, Jiamin Zhou, Ting Wu, Jingjing Qiu, Junxin Wang, Xicheng Song, Yan Sun

**Affiliations:** ^1^The 2nd Medical College of Binzhou Medical University, Yantai 264000, Shandong, China; ^2^Department of Otorhinolaryngology and Head and Neck Surgery, Yuhuangding Hospital Affiliated to Qingdao University, Yantai 264000, Shandong, China; ^3^Shandong Provincial Clinical Research Center for Otorhinolaryngologic Diseases, Yantai 264000, Shandong, China; ^4^Weifang Medical University, Weifang, Shandong, China

## Abstract

**Objective:**

Previous studies have yielded conflicting results regarding the association of coronavirus disease 2019 (COVID-19) with allergic rhinitis (AR). Data on AR prevalence in COVID-19 patients are limited. Consequently, whether AR is a harmful or protective factor for COVID-19 patients remains controversial. Therefore, we analyzed the relationship between COVID-19 and AR.

**Methods:**

We systematically searched PubMed, Embase, Cochrane, and Web of Science databases for studies published between January 1, 2020 and January 11, 2022. We included studies reporting the epidemiological and clinical characteristics of COVID-19 and its incidence in patients with AR. We excluded letters, case reports, literature review articles, non-English language article, and non-full-text articles. The raw data from these studies were pooled into a meta-analysis.

**Results:**

We analyzed the results of nine studies. The prevalence of AR in patients with COVID-19 was 0.13 (95% confidence interval [CI] 0.04–0.25), with an overall *I*^2^ of 99.77%, *P*=0.24. COVID-19 patients with AR are less prone to severe disease (odds ratio [OR] = 0.79, 95% CI, 0.52–1.18, *P*=0.25) and hospitalization (OR = 0.23, 95%CI, 0.02–2.67, *P* ≤ 0.0001) than patients without AR.

**Conclusion:**

Our data suggest that allergic rhinitis is a protective factor in patients with COVID-19.

## 1. Introduction

In December 2019, a novel coronavirus disease (COVID-19) emerged in Wuhan, China, and spread rapidly, leading the World Health Organization (WHO) to declare a pandemic for the first time in over 10 years. COVID-19 is a highly contagious and sometimes fatal disease, causing over 435 million cases to date [1]. COVID-19 leads to high utilization of medical resources that include nucleic acid testing, hospitalization, and intensive care. Determining which clinical factors place patients at high or low risk for severe COVID-19 is of great significance and can aid clinical decision-making [2]. Allergic rhinitis (AR) is a common chronic disease and often occurs in conjunction with combined airway disease, with an incidence of about 16.7% [3]. Whether AR acts as an independent risk factor for COVID-19 infection, severity, and hospitalization remains controversial [4].

Limited evidence suggests that AR exerts a protective effect against COVID-19 infection [4] and may reduce its severity [5]. On the contrary, a national cohort study in Korea showed that AR increased COVID-19 susceptibility and severity [4]. It has also been argued that AR is COVID-19 susceptibility and severity [6]. Chhiba et al. [7] reported that AR was not associated with an increased risk of hospitalization in patients with COVID-19.

Consequent to the conflicting findings of the aforementioned report, the objective of this study was to evaluate whether AR is a significant risk factor for COVID-19 infection, severity, and hospitalization. Such a determination may indicate the value of a history of AR as a prognostic indicator to facilitate clinical decision-making.

## 2. Methods

### 2.1. Search Strategy and Selection Criteria

In this meta-analysis, we searched PubMed, Embase, Cochrane Library, and Web of Science databases for articles published from January 1, 2020 to January 11, 2022. The electronic search was conducted using the strategy as follows: (1) “COVID-19”, OR “SARS-CoV-2,” OR “coronavirus disease 2019”; and (2) “allergic rhinitis”, OR “rhinitis” OR “allergy” OR “atopic”. Additional articles were retrieved by screening the list of references included in the study. The literature search was limited to articles published in English. Studies investigating the epidemiological and clinical features of COVID-19 were eligible.

### 2.2. Exclusion Criteria

Three reviewers (Cong Xu, He Zhao, and Yuwan Song) excluded studies that did not describe AR and COVID-19; studies that did not evaluate potential epidemiologic relationships between AR and COVID-19, studies that explored associations between COVID-19 and allergic diseases (AR and asthma as a composite variable); and studies that assessed the relationship between COVID-19 and one symptom of AR (e.g., sneezing). Letters, case reports, literature review articles, non-English language and non-full-text articles (e.g., editorials or congressional summaries) were excluded. EndNote (version X9.0) was used to manage records and exclude duplicate records.

### 2.3. Data Extraction

Three reviewers (Cong Xu, He Zhao, and Yuwan Song) independently screened the titles and abstracts of potential studies. Conflicts were resolved through discussion. We then independently read full-text articles to identify studies that met the inclusion criteria and carefully reviewed the reference lists from all identified studies and reviews for inclusion. For each study, the following data were extracted: the name of the first author; the country in which the study was conducted; cohort size; numbers of participants in severe and nonsevere disease groups; and numbers of participants in the inpatient and noninpatient illness groups.

The quality of studies in each was evaluated using the Newcastle-Ottawa Scale (NOS) by three reviewers (Cong Xu, He Zhao, and Yuwan Song). A total score of ≥7 indicated a high-quality study, whereas a total score of <7 was considered a low-quality study. Five factors (risk of bias, imprecision, inconsistency, indirectness, and publication bias) may cause a rating of the quality of evidence [8].

We conducted a meta-analysis on the prevalence rate of allergic rhinitis in patients with COVID-19 and calculated the combined prevalence rate with a 95% confidence interval (CI). The odds ratio (OR) was used to describe the relationship between the number of critically ill patients and inpatients with COVID-19 (dependent variable) and antecedent AR (independent variable). Due to heterogeneity within and between studies, we used a random effects model to estimate AR prevalence and calculated pooled ORs using Stata data and the Review Manager (version 5.3) tools analysis tool. The random effects model was used to estimate the mean effect and its accuracy, as it would provide a conservative estimate of the 95% CI. We used forest plots to represent the data and tested for between-study heterogeneity using the *I*^2^ statistic and *I*^2^ values >50% to indicate significant heterogeneity. We defined severe COVID-19 as cases requiring mechanical ventilation, vital life support, requiring intensive care unit admission, or ending in death. Funnel plots were used to assess publication bias.

## 3. Results

**Figure 1:**
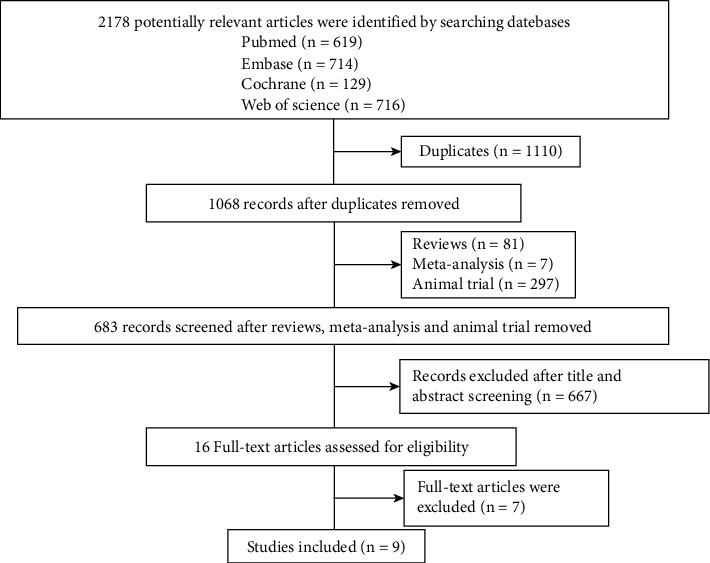
Flow diagram of study identification.

The initial search yielded 2178 potentially relevant papers, of which 1110 duplicates, 87 reviews, 297 animal experiments, and seven meta-analyses were excluded in the first screening of titles and abstracts. A total of 666 articles were excluded after the title and abstract screening. 16 papers met the inclusion criteria. After a more careful full-text review, seven additional papers were excluded for having incomplete data. Consequently, nine studies were included (Figure 1).

The nine studies included a total of 294,622 patients. Two studies were from the USA, two were from China, one from Turkey, one from Britain, one from Iran, and one from South Korea. The controls were patients without AR. In terms of study design, two articles (Jianjun Ren and Amirhossein Darabi) were prospective cohort studies, and the others were retrospective observational studies (Table 1). Two (22.2%) articles each had total NOS scores of 6, 8, and 9 and three (33.3%) articles had a score of 7 (Table 2). In addition, these studies have excluded the effects of comorbidities, especially respiratory.

### 3.1. Prevalence of Allergic Rhinitis in Confirmed COVID-19 Cases

**Figure 2:**
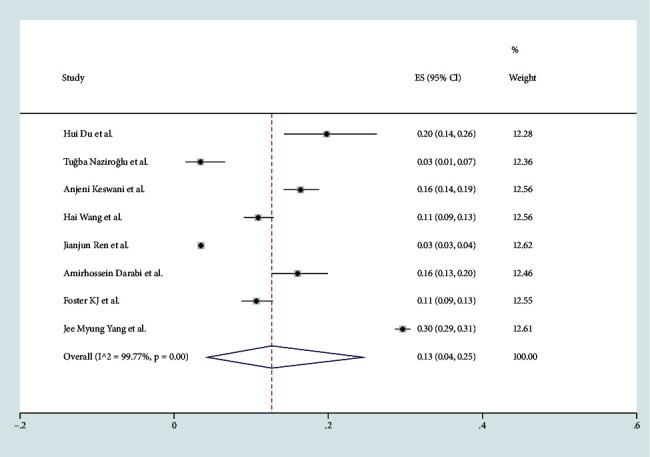
Meta-analysis of AR in COVID-19 cases of stay forest plot.

**Figure 3:**
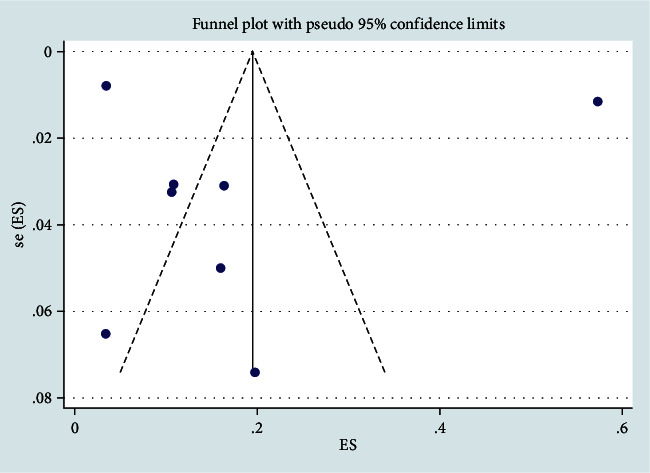
Meta-analysis of AR in COVID-19 cases of stay funnel plot.

The nine studies included 3,341 patients with AR, and the authors reported a total of 27,196 COVID-19 cases. The combined prevalence of AR in COVID-19 patients was 0.13 (95% CI, 0.04–0.25) (Figure 2). There was a high level of heterogeneity among the included case series (*I*^2^ = 99.77%; *P* ≤ 0.0001) (Figure 3).

### 3.2. Disease Severity and Hospitalization Rates among COVID-19 Patients with and without AR

**Figure 4:**
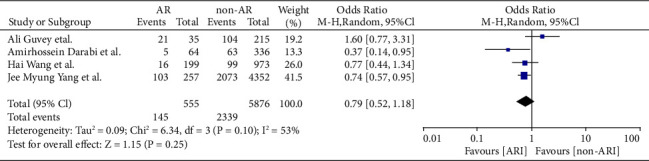
Incidence of severe COVID-19 with and without AR of stay forest plot.

**Figure 5:**
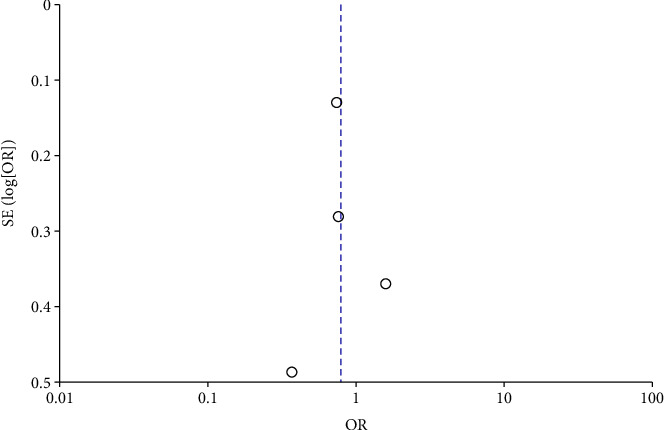
Incidence of severe COVID-19 with and without AR of stay funnel plot.

**Figure 6:**
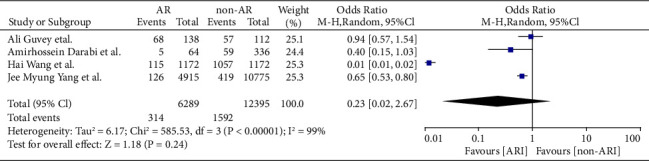
Incident hospitalization with COVID-19 with and without AR of stay forest plot.

**Figure 7:**
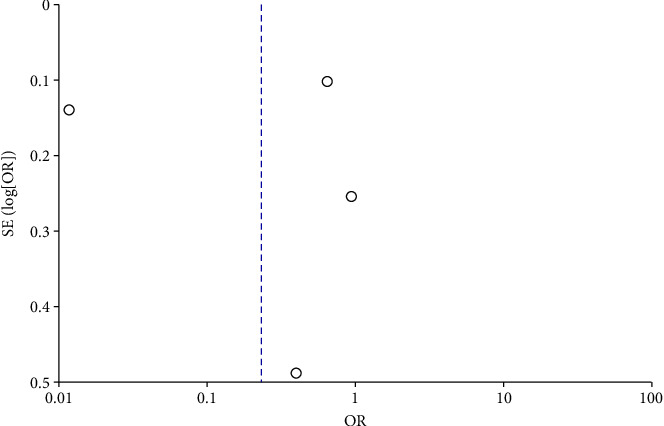
Incident hospitalization with COVID-19 with and without AR of stay funnel plot.

COVID-19 cases were classified as severe or nonsevere in only four of the nine studies. Reports on four studies involving 2,484 patients with AR contained COVID-19 data. Only three of the nine articles (involving 1,906 patients with AR) detailed the number of patients hospitalized. The meta-analysis showed that patients with AR have a lower risk of severe COVID-19 than COVID-19 patients without AR (odds ratio [OR] = 0.79, 95% CI, 0.52–1.18, *P*=0.25), which had less heterogeneity (*I*^2^ = 53%; *P*=0.1) (Figures 4 and 5). Moreover, patients hospitalized for COVID-19 were 0.23 times less likely to have comorbid AR (OR = 0.23, 95% CI, 0.02–2.67, *P*=0.24) (Figures 6 and 7). There was a high degree of heterogeneity (*I*^2^ = 99%; *P* < 0.00001).

## 4. Discussion

Our pooled analysis of published studies to date indicates that AR is considered comorbidity associated with reducing severity and hospitalization rates for COVID-19 patients.

The nasal cavity expression of ACE2 is abundant in patients with COVID-19 and acts as the cellular receptor that severe acute respiratory syndrome coronavirus 2 (SARS-CoV-2) uses to enter host cells [13]. Furthermore, SARS-CoV-2 dissemination also relies on the cellular serine protease TMPRSS2, which is also essential for the transmission of several clinically relevant viruses that include other beta-coronaviruses and the influenza A virus [14–17]. Kimura et al. [18] demonstrated that TMPRSS2 was elevated in the nasal and airway epithelial cells of AR patients, suggesting that AR patients are more susceptible to infection. Hoffmann et al. showed that host cell entry of SARS-CoV-2 can be partially blocked by a clinically proven inhibitor of TMPRSS2, which is employed by SARS-CoV-2 for S protein priming [14, 19]. However, according to the report, ACE2 plays a critical role in the development of COVID-19 and consequent lung injury [14, 20]. Some case studies have identified risk factors for serious illness, such as age, gender, hypertension, and diabetes, which can reduce ACE2 expression in vivo [21–24]. Nasal epithelial cells from participants with AR demonstrate lower ACE2 expression than healthy individuals [6, 18]. Cat allergens can significantly reduce ACE2mRNA expression in nasal brush samples from adult patients with AR caused by cat hypersensitivity. ACE2 gene expression was decreased in nasal and bronchial epithelial cells of AR patients, which reduces susceptibility to infection [6, 18]. Taken together, the results of these studies may provide a convincing physiological explanation of our finding that allergic rhinitis is a protective factor in patients with COVID-19.

Some studies suggest that AR drugs protect against the development of severe COVID-19 and patients taking these drugs are no more prone to SARS-CoV-2 infection [25–27]. This may be one reason that patients with AR and COVID-119 have milder pneumonia symptoms.

Histamine H1 receptor (H1 receptor) antagonists have immediate effects on sneezing and sniffle, which are used widely in the treatment of AR. Recently, many studies have shown that H1 receptor antagonists have direct antiviral activity against SARS-CoV-2 by interfering with the early steps of viral replication or by binding ACE2 [25, 28–30]. Patients taking these drugs had a significantly lower risk of SARS-CoV-2 infection [31]. In addition, treatment with H1 receptor antagonists and azithromycin prevented deterioration of lung inflammation in elderly patients with SARS-CoV-2 infection [32].

Montelukast, a cysteinyl leukotriene 1 receptor antagonist, may act as an antiviral agent by modulating innate and adaptive immunity [33]. It reduces mucus secretion from respiratory glands, affects lymphocyte activation and differentiation, and blocks the expression of inflammatory proteins in the lung by inhibiting type-2 T-helper (Th2) cytokines [interleukin (IL)-4, IL-5, and IL-13], especially in eosinophils [34, 35]. Levocetirizine, a third-generation antihistamine, and montelukast exhibit remarkable synergistic anti-inflammatory activity across a spectrum of signaling proteins, cell adhesion molecules, and leucocytes and eosinophil and neutrophil quantity and migration, which may prevent the progression of the disease from mild to moderate to severe and reduce both morbidity and mortality [36].

Th2 cytokine inhibitors reduce AR symptoms by inhibiting the production of Th2 cytokines, which are critically important in the pathogenesis of AR [37]. Poddighe and Kovzel considered that patients with COVID-19 taking such agents (omalizumab, anti–IL-5 biologics, and dupilumab) had milder or even no symptoms [38]. This finding is supported only by case reports and series; further studies, including those with case-control designs, are needed.

As with any meta-analysis, our study is susceptible to the limitations of the original studies, which may include design bias, selection bias, and residual confounding [39]. Due to these limitations, it is almost impossible to determine whether there were comorbidities other than allergic rhinitis in the COVID-19 patients. Advanced age, cardiovascular disease, and diabetes are associated with increased COVID-19 severity [23, 24]. In a systematic review of the nine articles, we found that only five had a positive comparison of outcomes between AR and non-AR patients. The results of our meta-analysis were heterogeneous in terms of sensitivity analysis, and a detailed analysis of forest plots showed that none of the included studies reported statistically significant differences between the two groups.

## 5. Conclusions

Our results suggest that COVID-19 incidence, severity, and risk of hospitalization are reduced in patients with AR. These findings strongly suggest that AR can be regarded as a protective factor and prognostic indicator in patients with COVID-19. This association may enhance our understanding of COVID-19 pathogenesis and provide a novel indicator for clinical decision support. Larger studies are needed to confirm these findings.

## Figures and Tables

**Table 1:** Summary of findings table.

Study	Country	Sample size	COVID-19 severe symptoms		COVID-19 hospitalization	
			AR	Non-AR	AR	Non-AR
Jee et al. [9]	China	5219,959	103 (257)	2073 (4352)	—	—
Hai et al. [6]	China	1172	16 (119)	99 (973)	115 (1172)	1057 (1172)
Amirhossein D et al. [5]	Iran	400	5 (64)	63 (336)	5 (64)	59 (336)
Ali [1]	Turkey	250	21 (35)	104 (215)	68 (138)	57 (112)
Jianjun et al. [4]	China	70557	—	—	126 (4915)	419 (10775)
Hui et al. [10]	China	182	—	—	—	—
Tuğba, Aksu. [11]	Ankara, Turkey	235	—	—	—	—
Anjeni et al. [2]	USA	2013	—	—	—	—
Foster et al. [12]	USA	1013	—	—	—	—

**Table 2:** Newcastle-Ottawa Scale scores for the included articles.

Study	Selection	Comparability	Outcome	Total scores
Jee et al. [9]	★★★★	★★	★★★	9
Hai et al. [6]	★★★	★★	★★	7
Amirhossein et al. [5]	★★★	★	★★★	7
Ali [1]	★★★★	★★	★★★	9
Jianjun et al. [4]	★★★★	★★	★★	8
Hui et al. [10]	★★★	★	★★★	7
Tuğba, Aksu. [11]	★★★★	★	★	6
Anjeni et al. [2]	★★★	★★	★★★	8
Foster et al. [12]	★★★	★★	★	6

## Data Availability

The data that support the findings of this study are available from the corresponding author upon reasonable request.
